# Bariatric Surgery and the Risk of Cerebrovascular Events: a Meta-analysis of 17 Studies Including 3,124,063 Subjects

**DOI:** 10.1007/s11695-022-06244-0

**Published:** 2022-09-21

**Authors:** Zixin Cai, Qirui Zhang, Yingling Jiang, Wei Liu, Jingjing Zhang

**Affiliations:** 1 National Clinical Research Center for Metabolic Diseases, Metabolic Syndrome Research Center, Key Laboratory of Diabetes Immunology, Ministry of Education, Department of Metabolism and Endocrinology, The Second Xiangya Hospital of Central South University, Changsha, 410011 Hunan China; 2 Department of General Surgery, The Second Xiangya Hospital, Central South University, Changsha, 410011 Hunan China; 3 Department of Biliopancreatic and Metabolic Surgery, The Second Xiangya Hospital, Central South University, Changsha, 410011 Hunan China; 4 Department of Metabolism and Endocrinology, Zhuzhou Central Hospital/Zhuzhou Hospital Affiliated to Xiangya School of Medicine, Central South University, Zhuzhou, 412007 Hunan China

**Keywords:** Bariatric surgery, Cerebrovascular, Meta-analysis

## Abstract

**Purpose:**

To perform a meta-analysis of the literature to evaluate the prevalence of cerebrovascular comorbidities between patients undergoing bariatric surgery and those not undergoing bariatric surgery.

**Materials and Methods:**

Studies about the risk of cerebrovascular disease both before and after bariatric surgery were systematically explored in multiple electronic databases, including PubMed, Web of Science, Cochrane Library, and Embase, from the time of database construction to May 2022.

**Results:**

Seventeen studies with 3,124,063 patients were finally included in the meta-analysis. There was a statistically significant reduction in cerebrovascular event risk following bariatric surgery (OR 0.68; 95% CI 0.58 to 0.78; *I*^2^ = 87.9%). The results of our meta-analysis showed that bariatric surgery was associated with decreased cerebrovascular event risk in the USA, Sweden, the UK, and Germany but not in China or Finland. There was no significant difference in the incidence of cerebrovascular events among bariatric surgery patients compared to non-surgical patients for greater than or equal to 5 years, but the incidence of cerebrovascular events less than 5 years after bariatric surgery was significantly lower in the surgical patients compared to non-surgical patients in the USA population.

**Conclusion:**

Our meta-analysis suggested that bariatric surgery for severe obesity was associated with a reduced risk of cerebrovascular events in the USA, Sweden, the UK, and Germany. Bariatric surgery significantly reduced the risk of cerebrovascular events within 5 years, but there was no significant difference in the risk of cerebrovascular events for 5 or more years after bariatric surgery in the USA.

**Graphical abstract:**

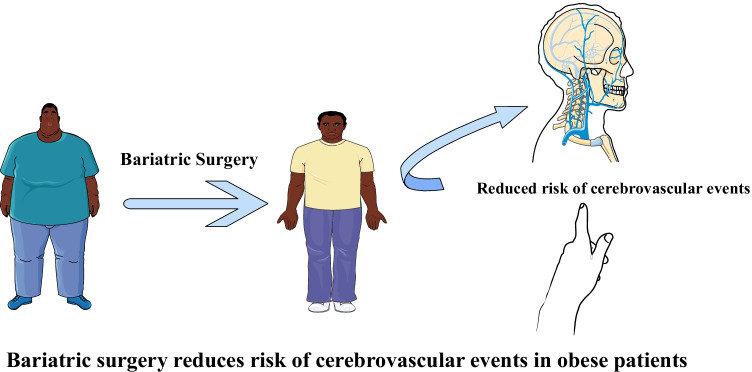

## Introduction

There has been an exponential rise in the global prevalence of obesity in recent decades. Obesity has a negative impact on life expectancy by increasing the risk of chronic health conditions, including cardiovascular disease, insulin resistance, sleep apnea, and other medical conditions [1]. There has been an exponential rise in bariatric surgery worldwide, with a significantly increasing rate every year. The NIH guidelines from 1991 advocated for bariatric surgery based on BMI and medical comorbidities. Patients suffering from obesity with a body mass index (BMI) > 40 kg/m^2^ or a BMI > 35 kg/m^2^ are recommended for bariatric surgery [2]. In recent years, the relationship between bariatric surgery and cerebrovascular events has received widespread attention. However, studies comparing bariatric surgery and cerebrovascular outcomes remain unclear. Thus, the aim of the current study was to conduct a meta-analysis to reveal the effects of bariatric surgery on cerebrovascular outcomes.

If bariatric surgery reduces the risk of cerebrovascular events, perhaps guidelines could be considered that recommend that overweight patients undergo bariatric operation to effectuate weight loss. Based on the above considerations, we performed a pooled analysis by integrating the results of previous works to obtain more robust and accurate estimates regarding the effect of bariatric surgery on cerebrovascular outcomes, which are vital to guide clinical management and counsel patients.

## Methods

### Search Strategy

The study was designed according to the PRISMA (Preferred Reporting Items for Systematic reviews and Meta-Analyses) checklist [3]. A systematic search was carried out on published studies for updates until May 2022 without language restriction in the PubMed, Web of Science, Cochrane Library, and Embase databases. We used the search terms “bariatric surgery” and “cerebrovascular disease.” Relevant articles were independently reviewed by two individuals.

### Study Selection

We included studies according to the following inclusion criteria: (1) articles reporting the relationship between bariatric surgery and cerebrovascular outcomes; (2) studies that included patients undergoing bariatric surgery; and (3) studies in which odds ratios (ORs) and their 95% confidence intervals (CIs) were collected or could be calculated from the information given. We excluded (1) reviews, editorials, correspondence, and meta-analyses; (2) studies with insufficient data; and (3) articles in non-English languages.

### Data Abstraction and Quality Assessment

We recorded the data from the selected studies using standard electronic sheets. The following information was extracted: the first author, the publication year, the study design, the source of the population, the proportion of men and women, the sample size, the postoperative period, the diagnostic criteria of the cerebrovascular comorbidities, and ORs with their 95% CIs.

The included studies were evaluated by two independent reviewers (ZC and QZ) using the quality assessment method, and disagreements were resolved via discussion. The Newcastle–Ottawa Scale (NOS) was used to assess the quality of the literature. Studies with NOS scores ≥ 7 were defined as high quality.

### Statistical Analysis

ORs were used to estimate the association between bariatric surgery and cerebrovascular comorbidities. Interstudy heterogeneity was assessed using *Q* value and *I*^2^ values*.* When the *I*^2^ value was greater than 50%, the random effects model was selected; otherwise, the fixed-effects model was used. For the qualitative interpretation of heterogeneity, *I*^2^ < 50% was considered to represent moderate heterogeneity, while *I*^2^ > 75% indicated extreme heterogeneity [4]. The potential for publication bias was evaluated graphically using both funnel plot inspection and Egger’s regression method for funnel plot asymmetry in the adjusted analyses of outcomes [5]. Sensitivity analysis was also performed to explore the stability of the results. Statistical analysis was conducted using Stata Statistical Software (version 12.0; STATA Corp., College Station, TX).

## Results

### Study Selection

**Fig. 1:**
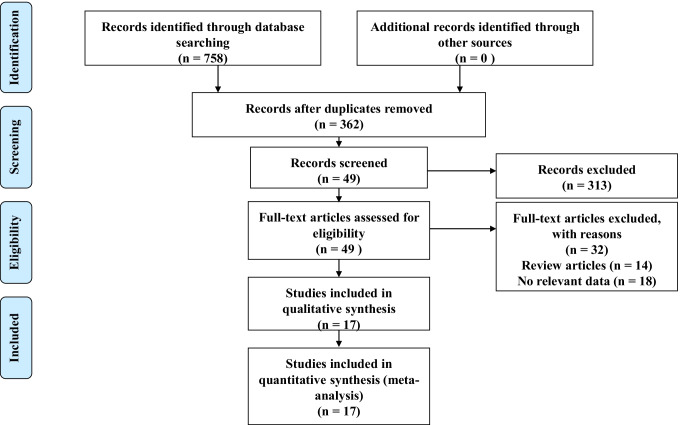
Flowchart of study selection

A total of 758 articles were retrieved via a primary search of the literature databases. After the removal of duplicates, 362 articles remained (Fig. 1). After screening both abstracts and titles, 49 studies were retrieved for full-text screening. Fourteen articles were excluded for review, and no relevant data about outcomes were identified in 18 articles. Ultimately, a total of 3,124,063 patients from 17 articles met the inclusion criteria and were included in the final meta-analysis [6–22]. The flowchart of the study selection process for the meta-analysis is presented in Fig. 1.

### Description of Included Studies

The basic characteristics of the included studies and quality evaluation are shown in Table 1. Our research included studies from seven different countries. The length of follow-up ranged from 1 to 14.7 years after bariatric surgery. The results of the literature quality assessment showed that the average score for the quality of the included studies was ≥ 7, and all of them were of high quality.

**Table 1:** Studies included in the meta-analysis

Number	Author	Year	Country	Design	Number	Age	Male percent	Diagnostic criteria of cerebrovascular time	Follow-up	NOS	OR	Ci 1	Ci 2
1	Jiewen Jin	2020	China	Retrospective	1,526,820	57.06 (0.08)	35.8% Male	Death	NA	9	0.71	0.62	0.81
2	Ali Aminian	2019	USA	Retrospective cohort	48,300	60.84 (10.59)	27.8% Male	Ischemic cerebrovascular accident	4 years	9	0.54	0.37	0.79
3	David P. Fisher	2018	USA	Cohort	20,235	49.5 (10.0)	24.1% Male	Ischemic stroke, hemorrhagic stroke, carotid stenting, or carotid endarterectomy	1 year	7	0.39	0.21	0.71
3	David P. Fisher	2018	USA						3 years		0.39	0.22	0.68
3	David P. Fisher	2018	USA						5 years		0.69	0.38	1.25
3	David P. Fisher	2018	USA						7 years		0.58	0.25	1.36
4	Michael D	2021	USA	Retrospective cohort	1,390,804	45 ± 9	20.8% Male	Ischemic stroke	1 year	8	0.54	0.47	0.61
4	Michael D	2021	USA						3 years		0.96	0.92	1
4	Michael D	2021	USA						5 years		0.78	0.65	0.9
5	Ali Aminian	2020	USA	Retrospective observational	7201	53.1 (44, 60.8)	32.2% Male	Cerebrovascular events (ischemic stroke, hemorrhagic stroke, or carotid intervention/surgery)	4.9 years	7	0.73	0.49	1.08
6	Erik Stenberg	2020	Sweden	Cohort	11,863	52.1 ± 7.46	34.2% Male	Subarachnoid hemorrhage, intracerebral hemorrhage, ischemic stroke, or acute cerebral event not specified as hemorrhage or ischemia registered in the NPR for in-hospital or outpatient care	4 years	8	0.81	0.63	**1.01**
7	Henry Buchwald	1998	USA	Retrospective	838	51	90.7% Male	Cerebrovascular events (cerebrovascular accidents and transient ischemic attacks)	5 years	7	1.3	0.91	1.85
8	Osama Moussa	2021	UK	Cohort	8424	50	20.1% Male	Cerebrovascular events: composite of acute ischemic stroke, transient ischemic event, non-traumatic subarachnoid hemorrhage, and non-traumatic intracranial hemorrhage	11.4 years	8	0.352	0.195	0.637
9	Osama Moussa	2020	UK	Cohort	3701	36 (29–44)	20.2% Male	Ischemic stroke	11.2 years	7	0.536	0.164	1.748
10	Thomas R	2017	USA	Retrospective	45,462	44.6 (11.3)	23.8% Male	Cerebrovascular accident/transient ischemic attack	NA	8	0.03	0.01	0.25
11	Shao-Lun Hung	2020	Germany	Retrospective	6265	32.39(8.63)	39.55% Male	Intracranial hemorrhage, epidural hemorrhage, ischemic stroke, and transient ischemic attack	3 years	**7**	0.162	0.073	0.36
12	Hedong Han	2019	China	Retrospective	24,534	60.94 (0.10)	38.6% Male	Acute ischemic stroke	NA	8	1.21	0.84	1.73
13	Maddalena Ardissino	2020	UK	Cohort	1186	49.63	34.91% Male	Ischemic stroke or transient ischemic attack or established cerebrovascular atherosclerosis	42.7 months	7	0.0227	0.0000946	5.451
14	STEFANO ROMEO	2012	Finland	Prospective	607	49 (6)	41% Male	Stroke	13.3 years	7	0.73	0.41	1.3
15	Lars Sjostrom	2012	Sweden	Prospective	2037		29.4% Male	Stroke	14.7 years	7	0.66	0.49	0.9
16	Christian Herder	2013	Finland	Prospective	3299	47.2(6)	30.9% Male	Stroke	10–13 years	7	0.998	0.921	1.083
17	Wenjing Tao	2014	UK	Cohort	22,487	NA	25% Male	Cerebrovascular disease (cerebral infarction or bleeding)	1 years	9	0.48	0.07	3.47

### Overall Analysis

**Fig. 2:**
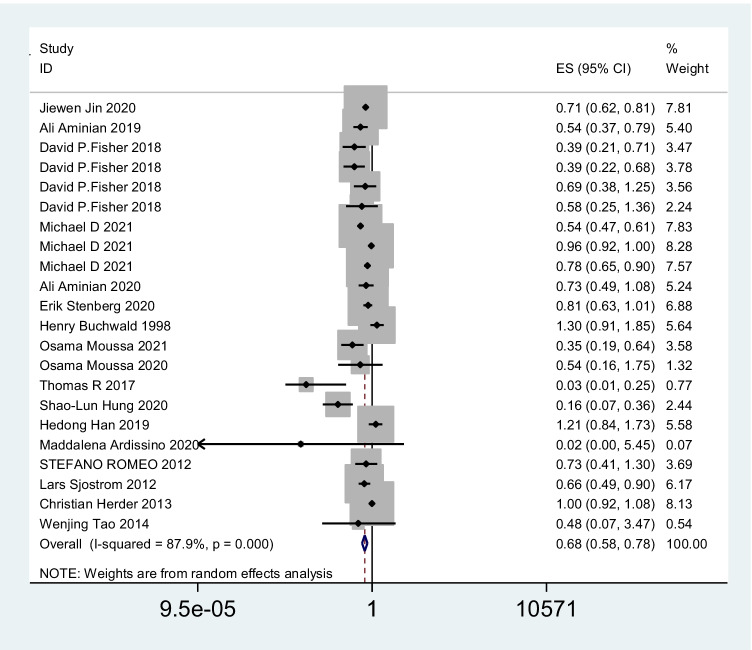
Forest plot comparing the odds of cerebrovascular risk between bariatric surgery and nonbariatric surgery patients

**Fig. 3:**
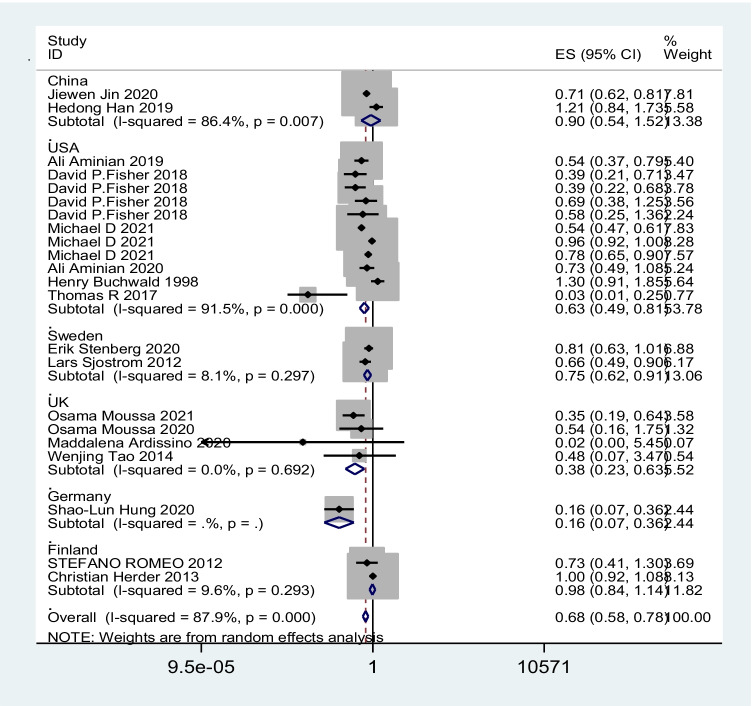
The odds of cerebrovascular risk between bariatric surgery and nonbariatric surgery patients stratified by country

**Fig. 4:**
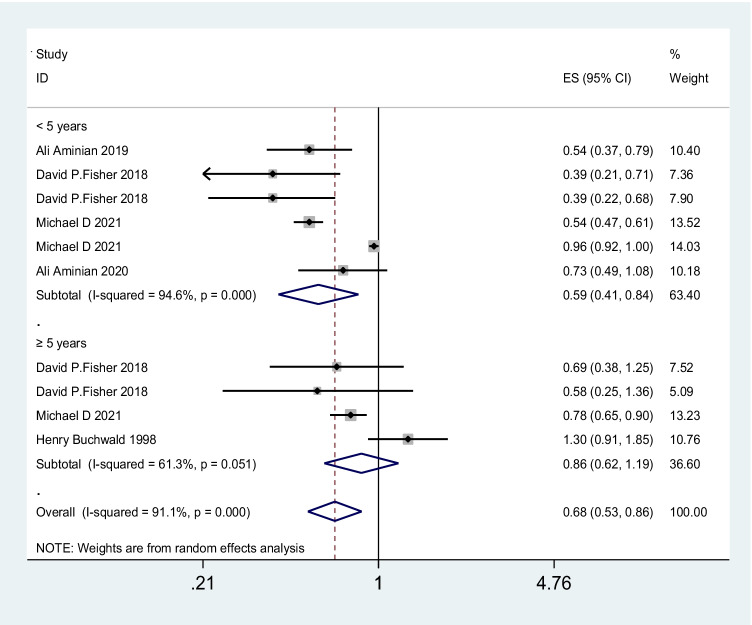
The odds of cerebrovascular risk between bariatric surgery and nonbariatric surgery patients stratified by follow-up time (< 5 years or ≥ 5 years)

**Fig. 5:**
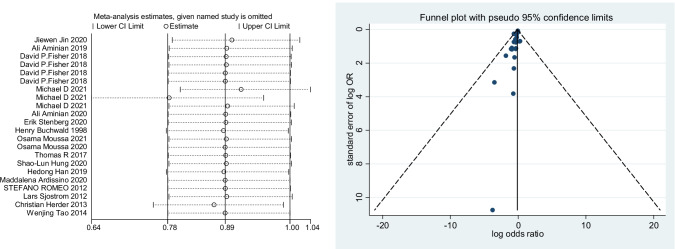
Publication bias funnel plots and sensitivity analysis

**Fig. 6:**
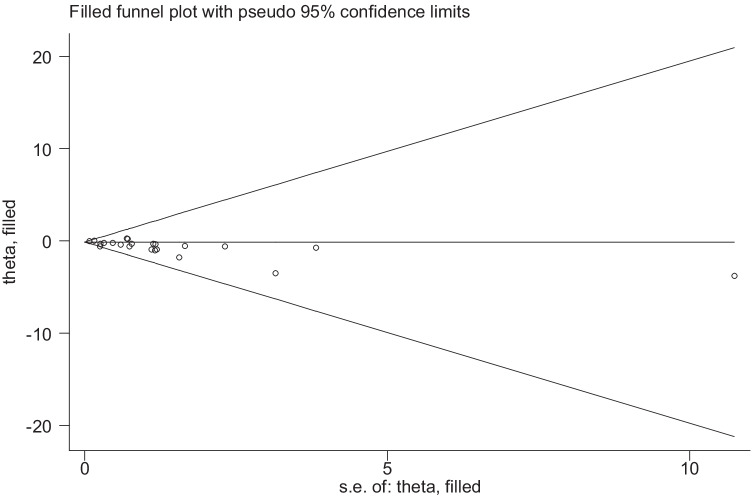
Trimmed and filled funnel plot of cerebrovascular risk and bariatric surgery

A total of 17 studies with 3,124,063 patients were pooled in the meta-analysis. The heterogeneity test (*I*^2^ value) revealed that the studies were heterogeneous (*I*^2^ = 87.9% > 50%); therefore, a random effects model was implemented for the analysis. As a result, the meta-analysis showed that the surgery group had a lower risk of cerebrovascular events than the no-surgery group (OR = 0.68; 95% CI 0.58–0.78) (Fig. 2). To explore the sources of heterogeneity, subgroup analyses were conducted based on country. The results of our meta-analysis show that bariatric surgery was associated with decreased cerebrovascular event risk in the USA (OR 0.63; 95% CI 0.49 to 0.81; *I*^2^ = 91.5%), Sweden (OR 0.75; 95% CI 0.62 to 0.91; *I*^2^ = 8.1%), the UK (OR 0.38; 95% CI 0.23 to 0.63; *I*^2^ = 0%), and Germany (OR 0.16; 95% CI 0.07 to 0.36) but not in China (OR 0.90; 95% CI 0.54 to 1.52; *I*^2^ = 86.4%) or Finland (OR 0.98; 95% CI 0.84 to 1.14; *I*^2^ = 9.6%) (Fig. 3). There was no significant difference in the incidence of cerebrovascular events compared with those without bariatric surgery after bariatric surgery for more than or equal to 5 years (OR 0.86; 95% CI 0.62 to 1.19; *I*^2^ = 61.3%), but the incidence of cerebrovascular events within 5 years after bariatric surgery was significantly lower than that of non-surgery patients in the USA population (OR 0.59; 95% CI 0.41 to 0.84; *I*^2^ = 94.6%) (Fig. 4). The sensitivity and publication bias analyses are displayed in Fig. 5. The results of the sensitivity analyses indicated that the meta-analysis had low sensitivity and that the overall results were robust and stable. The funnel plot displayed a symmetrical and funneled shape and Begg’s regression test (*p* > 0.05) suggested the absence of publication bias. In addition, the pooled results of bariatric surgery and cerebrovascular events were reliable after applying the trim and fill approach (Fig. 6).

## Discussion

### Association Between Bariatric Surgery and Cerebrovascular Events

This study evaluated 17 studies involving 3,124,063 individuals from a broad range of populations. Overall, the results from our study indicated that people with bariatric surgery had decreased odds of cerebrovascular comorbidities compared to nonbariatric surgery controls. Bariatric surgery significantly reduced the risk of cerebrovascular events within 5 years, but there was no significant difference in the risk of cerebrovascular events for 5 or more years after bariatric surgery in the USA. Weight loss after bariatric surgery is difficult to predict. Weight gain after bariatric surgery demonstrates the chronic and progressive nature of obesity. Therefore, follow-up after bariatric surgery is critical and requires a team approach for long-term benefits after bariatric surgery. This finding was consistent across most countries.

### Underlying Mechanisms of Bariatric Surgery Effects on Cerebrovascular Events

To date, the effect of bariatric surgery on cerebrovascular events is still unclear. The significantly lower risk of cerebrovascular events in the bariatric surgery group was related to the following: dyslipidemia, hypertension, and diabetes [23, 24].

Bariatric surgery has been reported to reduce the thickness of the media wall and pulse wave velocity of patients with dyslipidemia and hypertension [25]. Bariatric surgery has also been reported to attenuate inflammatory responses in patients with atherosclerosis [26, 27]. With weight reduction being an important measure to improve hypertension, bariatric surgery may also reduce the need for antihypertensive medications, reducing the risk for the development of organ damage [28].

Glycemic control is associated with the microvascular and macrovascular complications of diabetes, and it is reasonable to hypothesize that the improvement in glycemic control may translate to improved cerebrovascular events in these patients. Several studies have validated the benefits of bariatric surgery in long-term weight management, with peak weight loss 2 years post-surgery and stable good glycemic control for up to 20 years [29].

Laparoscopic sleeve gastrectomy, gastric bypass, and duodenal switch can alter hormone levels, such as those of ghrelin or GLP-1 [30]. Moreover, GLP-1 has been reported to influence blood glucose levels by decreasing hepatic gluconeogenesis [31]. Ghrelin promoted the synthesis of liver glycogen [32], increased blood glucose, and inhibited insulin release [33], all of which underscore the important effect of ghrelin in modulating glucose metabolism.

### Strengths and Limitations

The present study has a number of strengths in terms of the following aspects. First, this is the first study to report a meta-analysis comparing bariatric surgery and cerebrovascular events, including the largest highly representative population in the relevant area. Second, we searched and collected articles from four comprehensive electronic databases (PubMed, Embase, Web of Science, and the Cochrane library) without any restriction date; therefore, we were able to retrieve as many relevant articles as possible from all over the world and avoid the impact of publication bias. Third, several approaches, including subgroup analysis, sensitivity analysis, and publication bias analysis, were applied to establish whether the results of the present meta-analysis are reliable. Our results remained constant among these analyses.

There are several shortcomings in this meta-analysis that warrant mentioning. First, although we performed a subgroup analysis, we did not find the source of heterogeneity. Some confounding factors did not have enough studies to conduct subgroup analysis. Second, most of the included studies were cross-sectional studies and the causal relationship between bariatric surgery and cerebrovascular events could not be identified; therefore, further prospective longitudinal cohort studies are needed. Finally, variability in the diagnostic criteria of cerebrovascular events may have contributed to the high heterogeneity.

## Conclusion

Our study demonstrates the benefit of bariatric surgery on the risk of cerebrovascular disease. Based on our meta-analysis, bariatric surgery was associated with a lower rate of cerebrovascular disease. The risk of cerebrovascular disease was significantly reduced in the USA, Sweden, the UK, and Germany and was not significant in China or Finland. It is advisable to monitor patients closely after bariatric surgery, especially those at a high risk of cerebrovascular disease after bariatric surgery.

## Data Availability

All data generated or analyzed during the present study are included in this published article.
